# A microfluidic device for the hydrodynamic immobilisation of living fission yeast cells for super-resolution imaging^☆^

**DOI:** 10.1016/j.snb.2013.10.002

**Published:** 2014-03-01

**Authors:** Laurence Bell, Ashwin Seshia, David Lando, Ernest Laue, Matthieu Palayret, Steven F. Lee, David Klenerman

**Affiliations:** a Cambridge Nanoscience Centre, 11 J J Thomson Avenue, Cambridge CB3 0FF, United Kingdom; b Department of Biochemistry, 80 Tennis Court Road, Cambridge CB2 1GA, United Kingdom; c Department of Chemistry, Lensfield Road, Cambridge CB2 1EW, United Kingdom

**Keywords:** Microfluidics, Single cell analysis, Hydrodynamic trapping, PALM imaging, Single molecule localisation microscopy

## Abstract

We describe a microfluidic device designed specifically for the reversible immobilisation of *Schizosaccharomyces pombe* (Fission Yeast) cells to facilitate live cell super-resolution microscopy. Photo-Activation Localisation Microscopy (PALM) is used to create detailed super-resolution images within living cells with a modal accuracy of >25 nm in the lateral dimensions. The novel flow design captures and holds cells in a well-defined array with minimal effect on the normal growth kinetics. Cells are held over several hours and can continue to grow and divide within the device during fluorescence imaging.

## 1 Introduction

Super-resolution microscopy is an optical imaging technique which can allow three dimensional mapping of fluorescent particles with accuracy far beyond the diffraction limit of visible light (∼250 nm) [1]. However, its use in imaging tagged molecules within living cells is currently restricted, by the need to hold the target cell very still over an extended period of time [2]. For immobilisation to occur there must be some form of interaction with the cell and various approaches to this have been described [3], [4], [5]. However, for realistic investigations it is important that the trapping method used permits normal growth and division of the cell to occur while protecting it from excessive shear forces. Hydrodynamic trapping addresses many of these problems to some extent, and has also been described before [6], [7], [8], [9], but adapting these techniques to reliably and automatically capturing and holding a large array of cells, in a suitably high density is challenging; capabilities which are ultimately necessary for a robust and user-friendly device suited to super-resolution microscopy. The maintenance of a healthy, normal growing environment (at least over the time span in which the cell is expected to be within the device) is critical [10], [11], [12], [13], [14]. This means that a way to control the exchange of nutrients and dissolved gases, in suitable media and waste, to and from the cells, needs to be found while holding the cell reliably in place long enough to locate, manipulate (if necessary), and image. This must all be done without excessive movement or damage to the cell if the integrity of the final data is to be reliable.

**Fig. 1:**
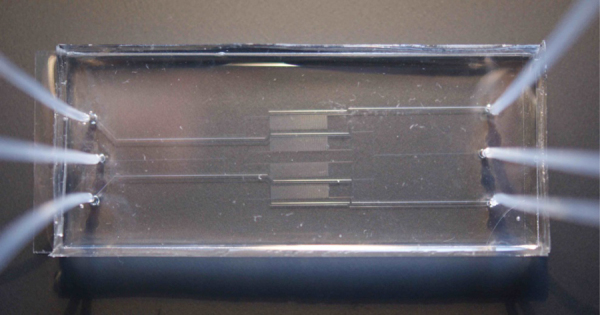
Image of the fabricated device showing external fluidic interconnects. The trapping layer is 52 mm from tip to tip, 10 mm wide and 5 μm thick, and is covered by a thicker control layer.

In order to increase the spatial precision in an imaging experiment, PALM sacrifices temporal resolution in order to achieve optimal results. PALM, therefore, places strict requirements upon the preparation of the samples, such as control over fluorescent contamination, avoidance of photo-conversion from ambient light sources and the need to keep the sample immobile in a well-defined space during the course of an imaging experiment. Attempts to image living cells over long time periods in growing conditions have been limited because of this; many requirements for healthy growing conditions (such as allowing for the free exchange of nutrient and waste around the cells), are in stark contrast to those for super-resolution imaging. Microfluidics can provide a solution to this stalemate, but to be of any practical use, the device must be simple and robust enough to be adopted by researchers who might have little experience with microfluidics, who in any case will want to focus on the microscopy and reduce the time and cost spent on sample preparation. In a recent review of Single Molecule Localisation Microscopy (SMLM), Patterson et al. listed this limitation as one of the main challenges facing these techniques, along with improvement of the instrumentation and the choice of fluorescent probe [15] (Fig. 1).

Fission yeast (*Schizosaccharomyces pombe*) is a genetically tractable eukaryotic organism that is used extensively in molecular and cell biology. They are a unicellular rod-shaped eukaryote, which typically measure ∼4 μm in diameter. Importantly, the dimensions of the long-axis of the cell (7–14 μm) is directly correlated to the point in the cell division cycle [16], allowing the interrogation of biological phenomena, such as DNA replication, repair, etc., within the context of the cell cycle. As the diameter of the cell does not change during replication *S. pombe* is especially useful for immobilisation with a hydrodynamic flow for super-resolution imaging in defined orientations and locations, over extended periods. While a number of previous groups have reported cell trapping and live cell studies in microfluidic devices, the particular requirements of PALM imaging in fission yeast place significant requirements on device design.

We present herein a device that was developed to meet the criteria necessary for PALM imaging of structures within living *S. pombe* cells over multiple cell cycles. These included hydrodynamically trapping cells within 1 μm of the imaging surface with minimal movement over extended time frames ranging from approximately 5–10 min to several hours, with control over the supply of fresh media as well as the temperature within the device.

## 2 Device design

The device utilises a ‘basin’-style design for hydrodynamic trapping [17], consisting of small trapping wells, each with a filtering ‘plug’ channel. This allows a slight pressure difference to build up when the plug is blocked by a cell. This holds the cell in place whilst allowing further flow to move past the site and so avoid blockage. The relative amount of flow through a plug compared to the flow around the basin determines the likelihood that a passing cell will be drawn into it – consequently the flow around the basin must be minimised to make the flow through dominant. This was done by forming rows of basins and gradually constricting the thickness of the main channel passing them to further increase the flow through the plugs. This also increases the resulting pressure difference when a cell is trapped, holding the cell more firmly in place. Excessive forces on the cell are reduced by deepening the well, removing it from the bulk of the flow so that the related stresses of the passing flow acts instead upon the media at the top of the basin. As the cells are not permanently immobilised, they may exhibit drift over extended time frames – to be an effective option for PALM imaging this drift must either be kept below the background noise level over the required time frames, or be regular such that it can be corrected for in the processing algorithm. The device contains three fluidic inlets feeding into four trapping regions all draining to a single, common drain (see Supplementary Information). The thicker control layer allows much higher flow volumes, providing temperature control by rapidly drawing heated or cooled water through the device.

The trapping region was designed with a zigzagging main channel forming 69 rows, each with 28 basins along it. Each basin is 22 μm wide with a central plug channel at the bottom, which is 2 μm wide and 35 μm long. This plug channel forms a shorter path to the next row. It is this reduction in path length, which increases flow through the plug and helps to draw a cell into the basin. Successful immobilisation of a cell removes this flow conduit, forcing later cells to continue past the basin and towards the next empty one. To improve the flow through each basin, the main channel gradually narrows along each row, decreasing in width by 1 μm after every basin, and returning to 50 μm at the beginning of each new row.

**Fig. 2:**
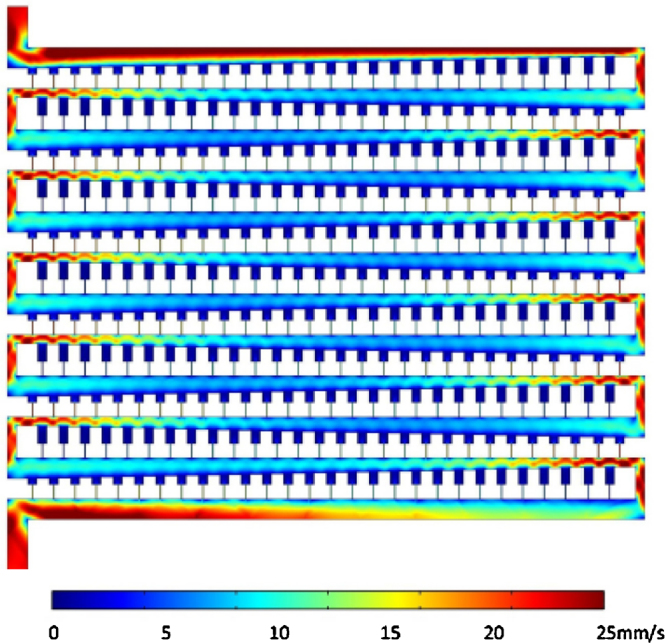
COMSOL simulation output showing the evolution of the flow velocity over 11 rows. The pressure difference is 1 kPa between the inlet, the outlet and the walls are assumed to exhibit full slip. The colour scale has been limited to values between 0 (dark blue) and 25 mm/s (scarlet). (For interpretation of the references to colour in this figure legend, the reader is referred to the web version of the article.)

As the main channel needs to maintain sufficient flow to carry cells and debris past the end of each row, the length of the rows was limited to 28 basins, leaving the main channel 22 μm wide at its thinnest point. The channels and trapping basins are designed to be 5 μm high to allow for *S. pombe* cells to be flushed through the device and simultaneously enable effective flow reduction across the trapping basin once a cell has been trapped. Fig. 2 shows a simplified 2D simulation of the fluid flow velocity through 11 rows of the trapping region. It was modelled using Comsol Multiphysics 4.0a, and shows that the fluid flow in the trapping region quickly forms a repeating pattern from row to row as it moves away from the inlet. This suggests that the design could therefore include more rows with little adverse effect on the efficacy of trapping in any one row, and so enable a higher number of trapping sites with dependably similar characteristics. This in turn means that a basin design which may have imperfect trapping efficacy can still be an effective device through sheer force of numbers: the present device includes four trapping regions, each with 1932 basins (giving 7728 trapping sites per device), so a design with trapping efficiency of only 1% still provides the likelihood of not only sufficient immobilised cells for manual PALM use, but also enough to allow automated sets of measurements to be carried out as well. With the aid of a mechanical optical stage in the PALM setup, it then becomes a straightforward matter to scan the sites using imaging software from above and automatically image a filled site from below when trapped cell is detected.

**Fig. 3:**
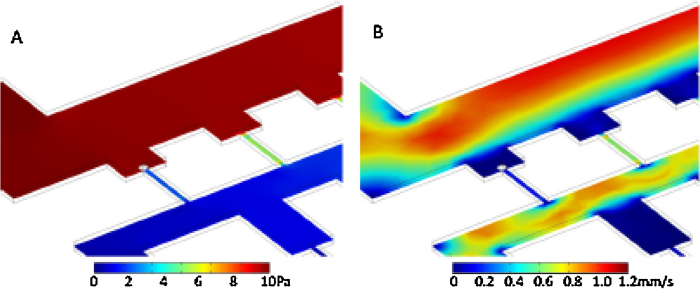
Simulation of the trapping of a cell. (A) Pressure. Flow through a plug channel is largely blocked when a cell enters, immediately setting up a large pressure difference which holds it firmly in place. (B) Flow velocity. The reduction in flow through the plug makes it less likely that another cell will enter the same well and become trapped.

Fig. 3 shows the change in pressure and flow velocity caused by a sphere 5 μm in diameter partially blocking the plug channel of the first available site. Fig. 3(A) shows the pressure, which holds the cell in place without stopping the passing flow in the main channel. Fig. 3(B) shows a much reduced (although non-zero) flow through the plug channel beneath the trapped cell, with a minor increase in the flow through the neighbouring basin. This suggests that the design tends towards exclusivity in trapping one cell per basin. The depth of the basin has little effect on its efficiency, as long as it is deep enough to shield the trapped cell from the main flow. This reduces any forces that might act to either move the cell or cause unnecessary shear stress on it – a feature which is lacking in many pre-existing similar cell trapping designs [17], [18].

**Fig. 4:**
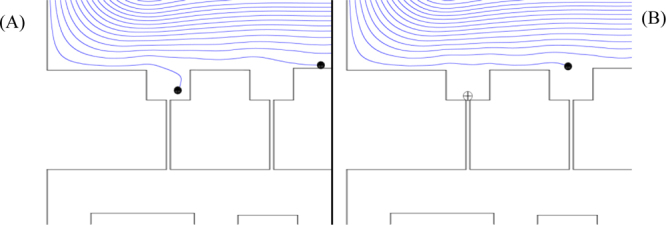
Simulated tracing of zero-mass particles through the first row of the device. (A) A particle entering the trapping region near the wall is quickly drawn into the first site, while others are pulled closer as they pass each empty site. (B) When the first site is already occupied, the same particle flows past it and enters the second site instead.

The same model was then simulated with a sample injection of massless particles to assess the likelihood of site exclusivity. 19 particles were equally spaced across the inlet and particle tracing was used to investigate the likely pathway followed by each one. Fig. 4 shows the difference made to these pathways when one site is blocked. Fig. 4(A) shows the proximal cell being drawn straight into the first vacant site and towards the plug channel, with the other cells gradually pulled towards the basins. In Fig. 4(B) the first site is occupied and a cell, which would have otherwise entered, is pushed instead on to the next basin.

The results obtained from numerical simulations are qualitatively consistent with an analytical formulation for fluid flow based on a Poiseuille model [19] indicating an approximately equal flow resistance for flow through the first trapping site relative to the wide bypass channel and an approximately linear incremental increase in relative flow resistance for flow through successive trapping sites situated further downstream along the channel. Peclet analysis of this design suggests that species transport can be dominated by either advection or diffusion, depending largely upon the flow velocity and species involved; in Fig. 3, Fig. 4, for example, the maximum flow velocity in the main channel is around 1 mm/s. The hydraulic diameter for this channel is around 10 μm (defined as twice the depth of the channel for wide, enclosed channels). This yields a Peclet number of around 5 for dissolved O_2_ (with a mass diffusion coefficient of roughly 2 × 10^−9^ m^2^/s in water) – this is similar for most ions and up to 10 or 100 times higher than for larger biological molecules (with typical mass diffusion coefficients between 10^−10^ and 10^−11^ m^2^/s. This means that most of the transport of these species is due to flow rather than diffusion in this case. Within the basins, however, the flow velocity is much lower than in the main channel, and this (coupled with the option of reducing or even stopping the flow through the device when desired) allows the user to switch instead to a diffusion-dominated regime.

## 3 Materials/methodologies

Master moulds were fabricated on clean 75 mm silicon wafers (supplied by Compart Technology Ltd., Peterborough, UK), and layers of SU-8 2005 (5 μm thick for the channel layer) or SU-8 2025 (100 μm thick for the control layer) were fabricated according to the procedure supplied by MicroChem, including a post bake at 200 °C for 5 min to further strengthen the polymer. A coating of 1H,1H,2H,2H perfluorodecyltrichlorosilane (FDTS, supplied by Alfa Aesar, Heysham, UK) was added to increase the durability and hydrophobicity of the master by placing it in a vacuum desiccator with an open vial containing a few drops of FDTS in hexane, and pumping down until the hexane boiled away. The desiccator was left under weak vacuum overnight to allow the gradual formation of a monolayer of FDTS. This coating did not appreciably increase the thickness of the plug channels, yet greatly improved the removal of cured PDMS, and remained undiminished after many mouldings.

To make a device, PDMS (Sylgard 184 Silicone Elastomer purchased from Dow Corning, Barry, UK) was prepared according to the manufacturer, poured onto the channel layer master mould and then spun at 3500 rpm for 60 s to reduce the thickness to about 20 μm. This thickness was chosen so that the channels were covered by around 15 μm of PDMS. The thicker control layer was poured to a thickness of 5–7 mm to make the device more robust and give better sealing around the tubing. Once cured, the control layer was trimmed and holes were punched for the temperature control flow using a 1.0 mm biopsy punch, before both it and the channel layer (still on the wafer mould) were activated in oxygen plasma for 10 s at 1.0 mbar in a Deiner Etcher. The two layers were then aligned and joined, and then the channel layer was trimmed around the thicker control layer and peeled off the mould as one piece. Inlet/outlet holes were punched, then a 20 mm × 60 mm borosilicate slide was cleaned in Piranha etch before it and the PDMS channels were activated again in oxygen plasma and joined. The device was then ready to be used immediately. However, because it was entirely dry and stable, no degradation was observed in devices that had been stored for six weeks before use. To prevent contamination during this time, Scotch tape was stuck to the top and bottom surfaces immediately after fabrication, and removed immediately prior to use.

## 4 Proof of concept experimental results

**Fig. 5:**
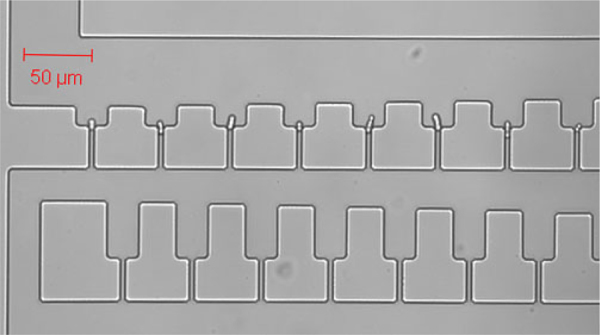
Confocal microscope image of single *S. pombe* cells held separately in the first row of a trapping region.

**Fig. 6:**
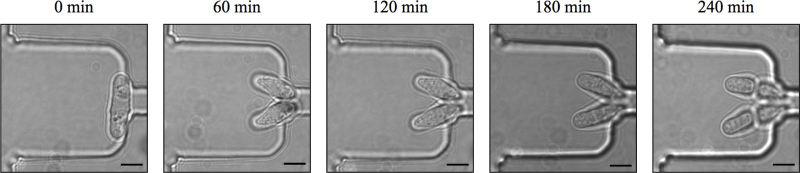
The device sustains normal growth and division of *S. pombe*. A single cell was immobilised and imaged at 60 min intervals with transmitted white light. Scale bar, 5 μm.

*S. pombe* cells were grown in YES media (0.5% yeast extract, 3.0% glucose, 200 mg/L adenine, histidine, leucine, uracil, lysine purchased from Sigma) at 30 °C to a density of roughly 10^7^ cells/mL, and then loaded into a 1.0 mL syringe and connected to the fluidic device via 1.09 mm (OD) polythene tubing. The cells were pumped through the device at a constant flow rate of 10 μL/h using a syringe pump. The device was imaged using a Zeiss LSM 510 Meta confocal microscope with a 532 nm laser light source, and the device behaviour was assessed. An image of early filling of the device with cells is shown in Fig. 5. In this image a single cell can be clearly seen in each of the sites in the first row, held in place by one end stuck in the top of a plug channel. These cells did not move from the basins until the fluid flow stopped, however, many gradually settled or grew deeper into their respective plug channels. To ensure proper cell growth heated water was then pumped through the control layer at a flow rate of 1 mL/min so that the glass slide of the device, where the cells were immobilised against, was maintained at 28 °C (±2 °C). Monitoring the temperature of the glass slide was carried out using an infrared laser thermometer (Fisher Scientific). Under these conditions *S. pombe* cell growth appeared normal and cells divided with an average cell cycle time of 2.5–3 h (Fig. 6 and Supplementary Fig. S-4) which is consistent with cell growth outside of the device.

## 5 PALM imaging

The centromeric histone H3 protein variant, Cnp1, is a good example of a mediator of epigenetic inheritance and an excellent candidate for SR fluorescence studies. Cnp1 proteins, appear as a punctate spot that divides as the cells progress through mitosis. A strain of *S. pombe* in which the endogenous cnp1 gene was N-terminally tagged with the mEos2 fluorophore [20] was grown overnight at 30 °C in minimal EMM media (Formedium, Norfolk, UK) supplemented with 2% glucose and 0.2 g/L each of histidine, uracil, leucine, lysine and adenine. For live cell imaging the cells were loaded into the device at a rate of 10 μL per hour and maintained at 28 °C (±2 °C) by pumping heated water through the control layer. For fixed cell samples, cells (2.0 × 10^7^) were resuspended in 1 mL of phosphate buffer (100 mM Na-Phosphate pH 7.5) containing 1% paraformaldehyde and incubated at room temperature for 20 min. Fixed cells were washed (3×) with 1 mL of phosphate buffer and the pellet resuspended in 20 μL phosphate buffer. One microliter was then overlaid onto an agarose pad sandwiched between two cover-slip slides.

### 5.1 Single-molecule fluorescence imaging

White light transmission and single-molecule fluorescence images were acquired with an Olympus IX71 inverted microscope equipped with an infinity-corrected oil immersion objective (Olympus UPlanApo TIRF, ×60, 1.49 NA) and detected on a 512 × 512 pixel EMCCD (Photometrics Evolve, EVO-512-M-FW-16-AC-110) at a rate of 33 ms per imaging frame. The general epifluorescence setup has been described previously [21], [22]; here the filters used were a dichroic mirror (Semrock, Di01-R488/561) and a long pass filter (Semrock, LP02-568RS-25) designed for imaging the photo-converted form of the mEos2 fluorophore. Images were acquired using 561 nm excitation light (Cobalt Jive, 0561-04-01-0100-300) at power densities of ∼4 kW/cm^2^ and the photo-conversion was performed using 405 nm laser light (Oxxius 405, LBX-405-100-CIA-PP) at power densities of ∼10 W/cm^2^. A pulse sequence was chosen to ensure the irreversible bleaching of single mEos2 molecules in the cluster prior to the next imaging cycle. Here 10 imaging frames were performed before a single activation pulse. This was repeated until no further fluorescence activation was observed (∼5000 frames). Laser intensity modulation was achieved using mechanical shutters (Prior, Optiscan III, V31XYZEF) and controlled using a bespoke script in the open source microscopy software μmanager [23].

**Fig. 7:**
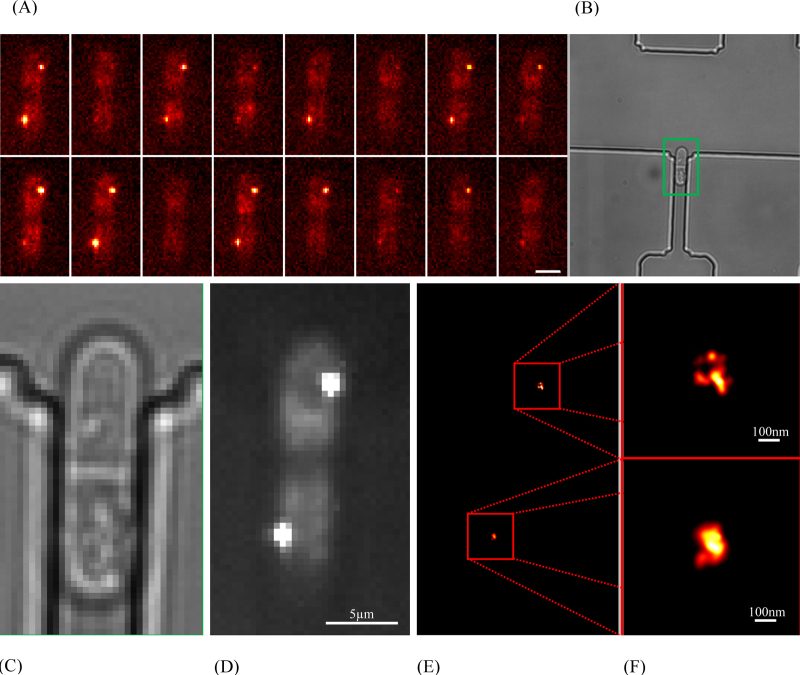
(A) Time montage of raw single image frames for a trapped *S. pombe* cell. (B) A cell was immobilised and imaged with transmitted white light. (C) A zoomed image where the septum is clearly visible, and the cell is nearing fission. (D) Epifluorescence image of the same cell showing the fluorophores clustered around the two separate centromeres. (E) Corresponding PALM image of the fluorophore positions. (F) Increased resolution image of the fluorophore centres showing the level of detail achieved.

Super-resolution images (Fig. 7) were obtained using image processing techniques detailed previously [24]. Briefly, for each 33 ms imaging frame, the position of a single emitter was determined by first approximating the point spread function as a 2D Gaussian function, then fitting the signal above background using the nonlinear least squares regression function (nlinfit) in MATLAB^®^ (MathWorks).

Finally, each single-molecule position was re-plotted using a custom macro written in ImageJ (http://rsb.info.nih.gov/ij/) as a 2D Gaussian profile defined by the measured integrated intensity and a width given by the statistical error (95% confidence interval) in localisation of the centre.

The acquisition lasted around 3 min, during which time 147 excitation cycles were run, capturing fluorescence data from 138 separate single molecules, with a modal localisation precision of 21 nm (the whole distribution is included as Supporting Material Fig. S5.). These values are consistent with other methods of immobilisation demonstrating that the device does not adversely affect the precision within the limits of detection when using fluorescent proteins such as mEos2. A representative localisation distribution for a PALM imaging taken outside the device on an agarose pad is included showing no significant difference.

In order to illustrate the compatibility of the device with large automated sample gathering, we demonstrate that the position, focal plane, and cellular length (of the *S. pombe* cells being studied) can all be automated using simple image processing techniques. Here computer control is used to evaluate the existence of a trapped cell, reposition the cell in the centre of the field of view, focus on it, and perform a PALM experiment. We envisage this being used for both large-scale data acquisition and in ‘smart’ imaging algorithms whereby only cells in an important but short-lived part of the division cycle (e.g. during mitosis) are to be imaged. This algorithm was also implemented in the open source microscopy platform μmanager [23] (the code is available on request) and screen shots and a description of the process are included as Supplementary Material (see Fig. S-6, Text S-1 and Movie S-1).

## 6 Conclusions

Normal growth and division of *S. pombe* cells in the microfluidic device has been demonstrated, with highly controlled and reversible immobilisation. The device permits the variation of media flow before, during and after super-resolution imaging. Trapped cells have shown a high probability of being immobilised at the entrance to the plug channel, forming a regular array 50 μm from the next site with an appreciable probability of site exclusivity. The device is readily replaceable, as the material and time costs for a single device are very low, and it is easy to fabricate and use. Finally, we show the possibility of using computer controlled automation to collect large sample sets.

The results presented here show that the described device meets or exceeds all of the necessary criteria for the hydrodynamic trapping of *S. pombe* cells for super-resolution imaging. It has shown no obstruction to the normal operation of the PALM instrumentation, allowing us to obtain images consistent with high quality single molecule super-resolution imaging of fluorescent proteins. Early experiments using the device have shown that the resolution currently attainable is not limited by the device, providing a powerful tool for future research. The results show a clear improvement over existing techniques for single cell immobilisation and imaging, as the described approach does not subject the cells to excessive heating or electric fields, and also presents a more ‘natural’ environment around the cells than is achieved by trapping them in a 3-dimensional gel, as well as providing far better spatial control over the trapping sites. Furthermore, this method improves upon cell adhesion techniques by maintaining a reversible, lower-impact contact between the device wall and the cell wall. The trapping method and microfluidic interface is extendable to other super-resolution microscopy setups.
