# Supplementary material for: A microfluidic device for the hydrodynamic immobilisation of living fission yeast cells for super-resolution imaging
Source: Sens Actuators B Chem. 2014 Mar 1;192:36–41. doi: 10.1016/j.snb.2013.10.002 (PMC4375559; doi:10.1016/j.snb.2013.10.002)
Supplement: Supplementary file 1 [file mmc1.doc]

**Supplementary Information**


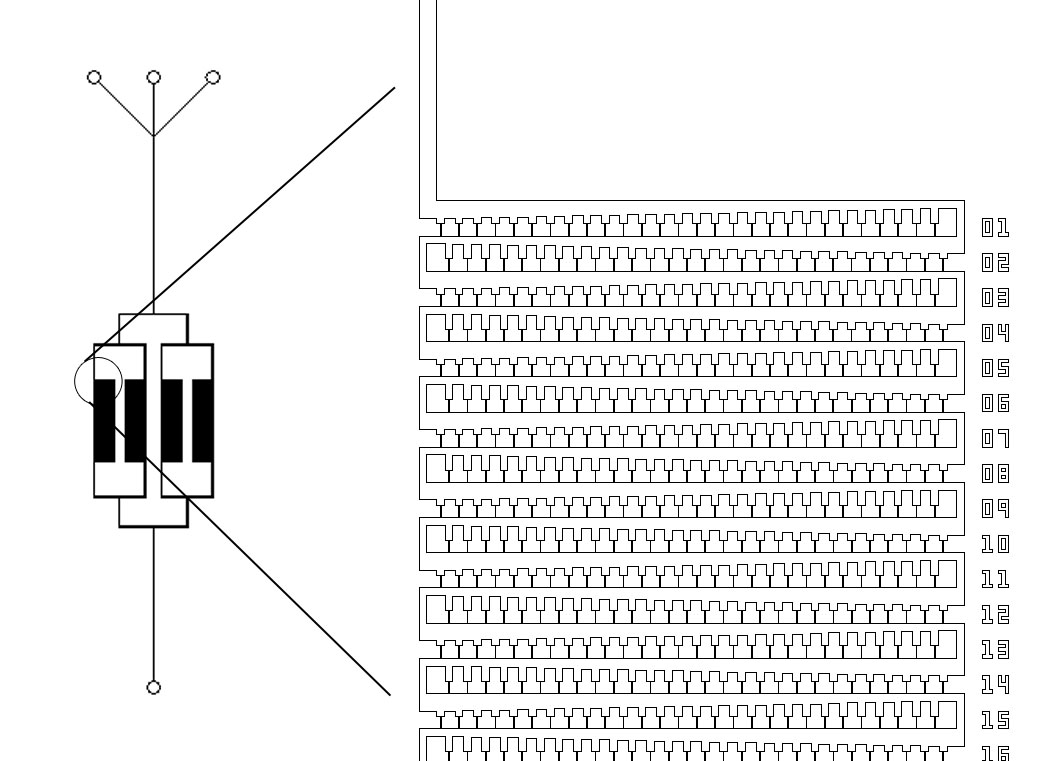


Figure S-1: Design overview. The device consists of three inlets feeding into four trapping regions and draining to a single, common drain. The trapping region was designed with a zigzagging main channel forming 69 rows, each with 28 basins along it. Each basin is 22 μm wide with a central plug channel at the bottom, which is 2 μm wide and 35 μm long.

(d)

(c)


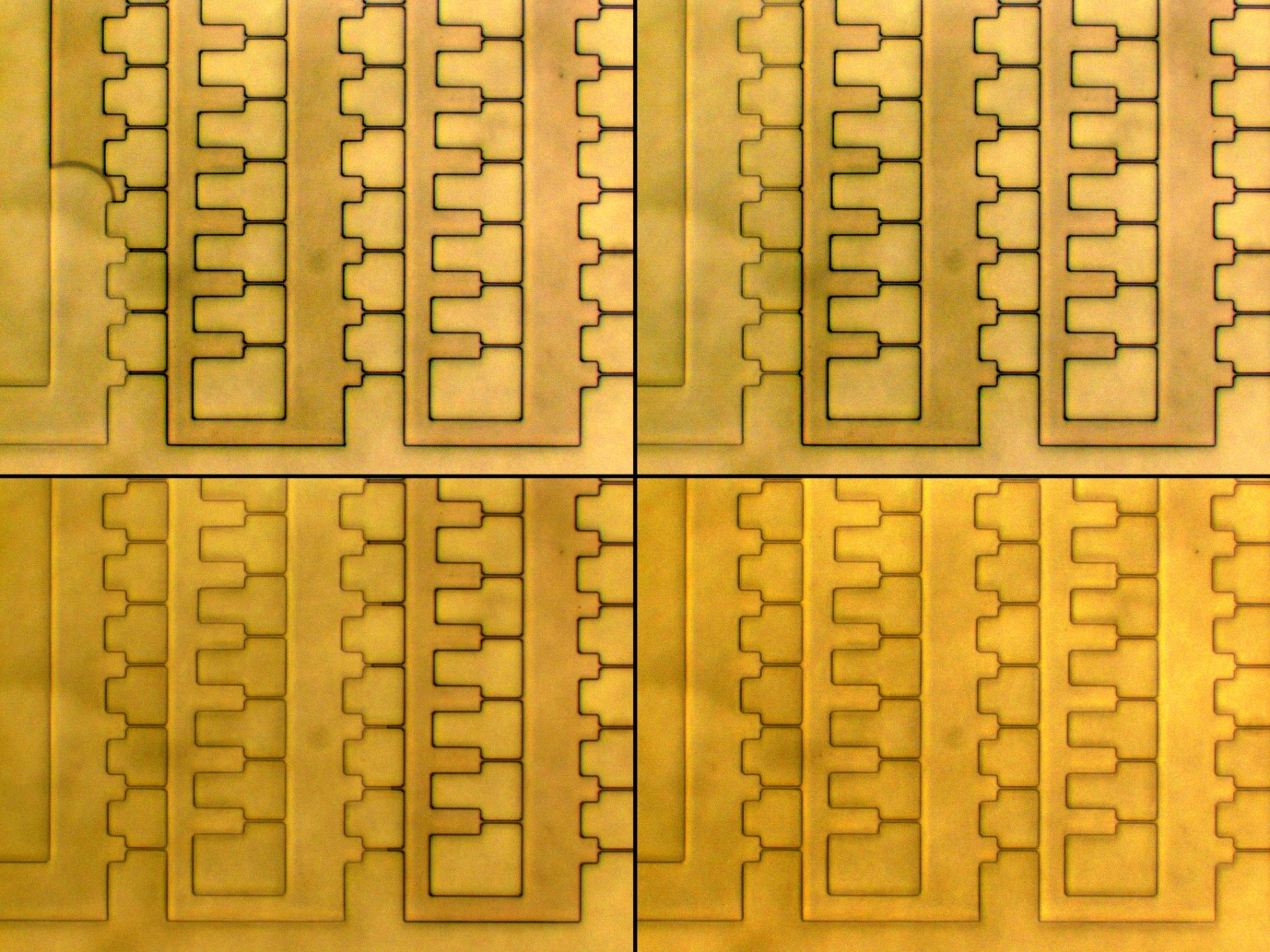


(b)

(a)

Figure S-2: Initial filling of the device with deionised water from the inlet at the bottom left corner. (a) Beginning to fill. (b) Capillary action draws the water through the plug channels. (c) Any bubbles formed evaporate quickly. (d) Device is filled and primed.

A

e

d

c

b

a


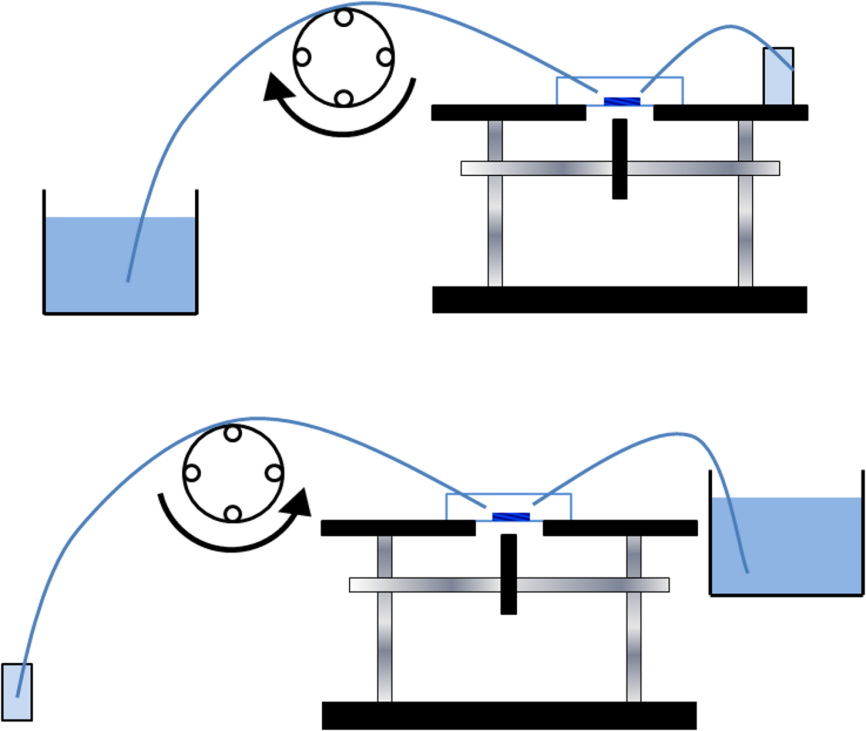


B


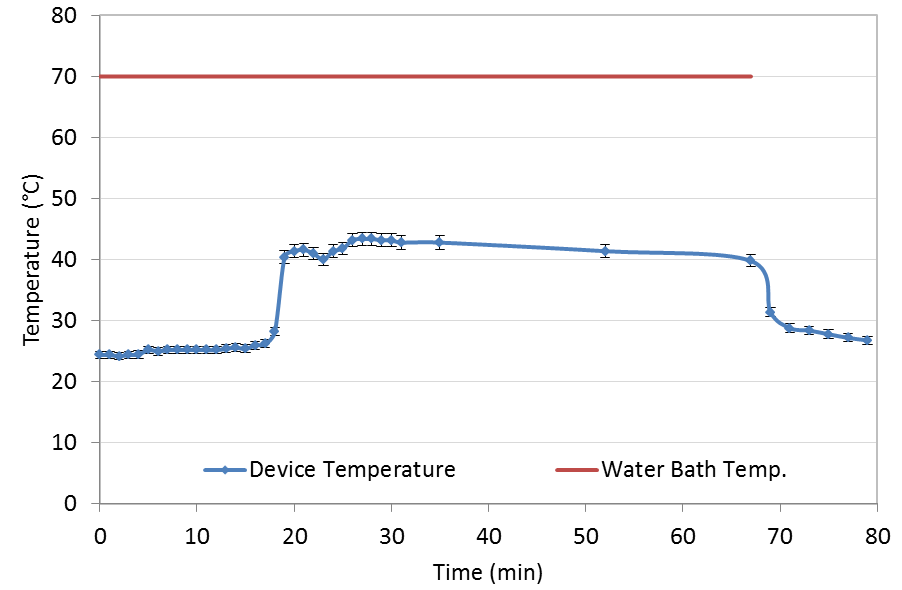


Figure S-3: A. Schematic showing temperature control for the microfluidic device. A peristaltic pump (a) draws heated water from a reservoir (b) through the device under test (c) into a waste reservoir (d). An IR thermometer (e) measures the temperature. B. The channels can be heated to above 40^o^C by passing water at 70^o^C through the temperature control layer.

Figure S-4: Overnight growth of *S. pombe* within a device under constant flow. This is a snapshot showing detail within rows 17-25 of 69 such rows built into the device. Seed’ cells which escaped the blockage became trapped further down the trapping region in random, sparse sites. Once immobilised, the cells stayed in position dividing through several generations.


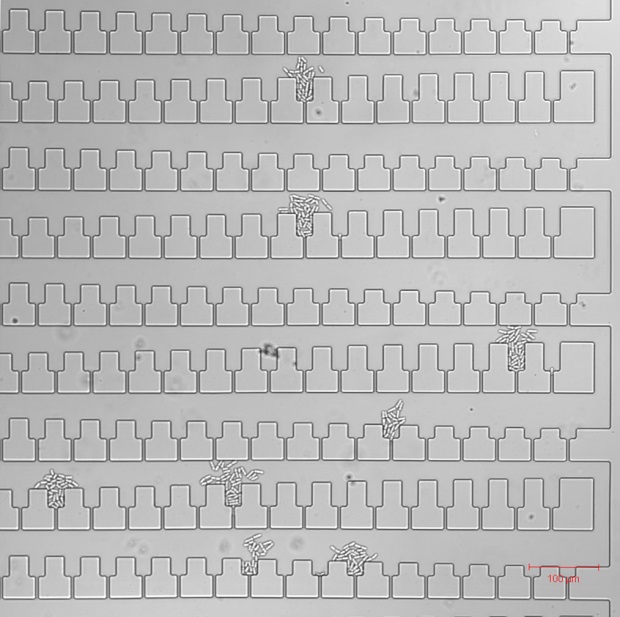

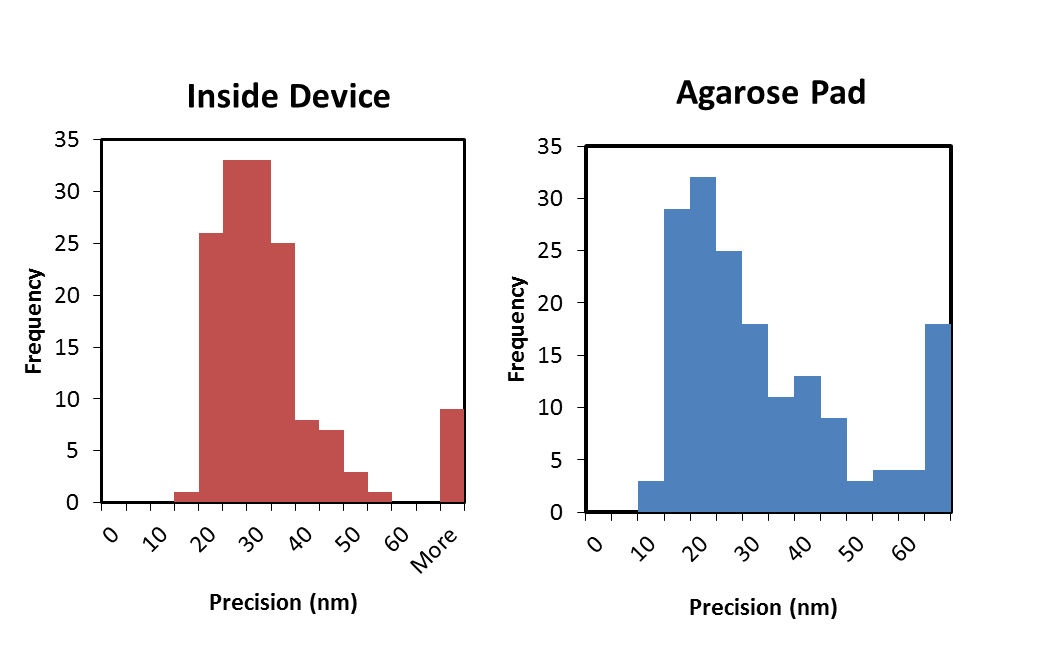


**Figure S-5:** Histogram of the localisation precision of single mEos2 fluorophores. A representative distribution of the localisation precision achieved in PALM imaging for an individual cell in either the microfluidic device (red) or in agarose pads (blue) is shown. There is no appreciable difference between data taken inside the microfluidic device and data taken outside. In these instances, the cell imaged inside the device had 138 localisations of Cnp1, while the cells outside the device on an agarose pad had 167.


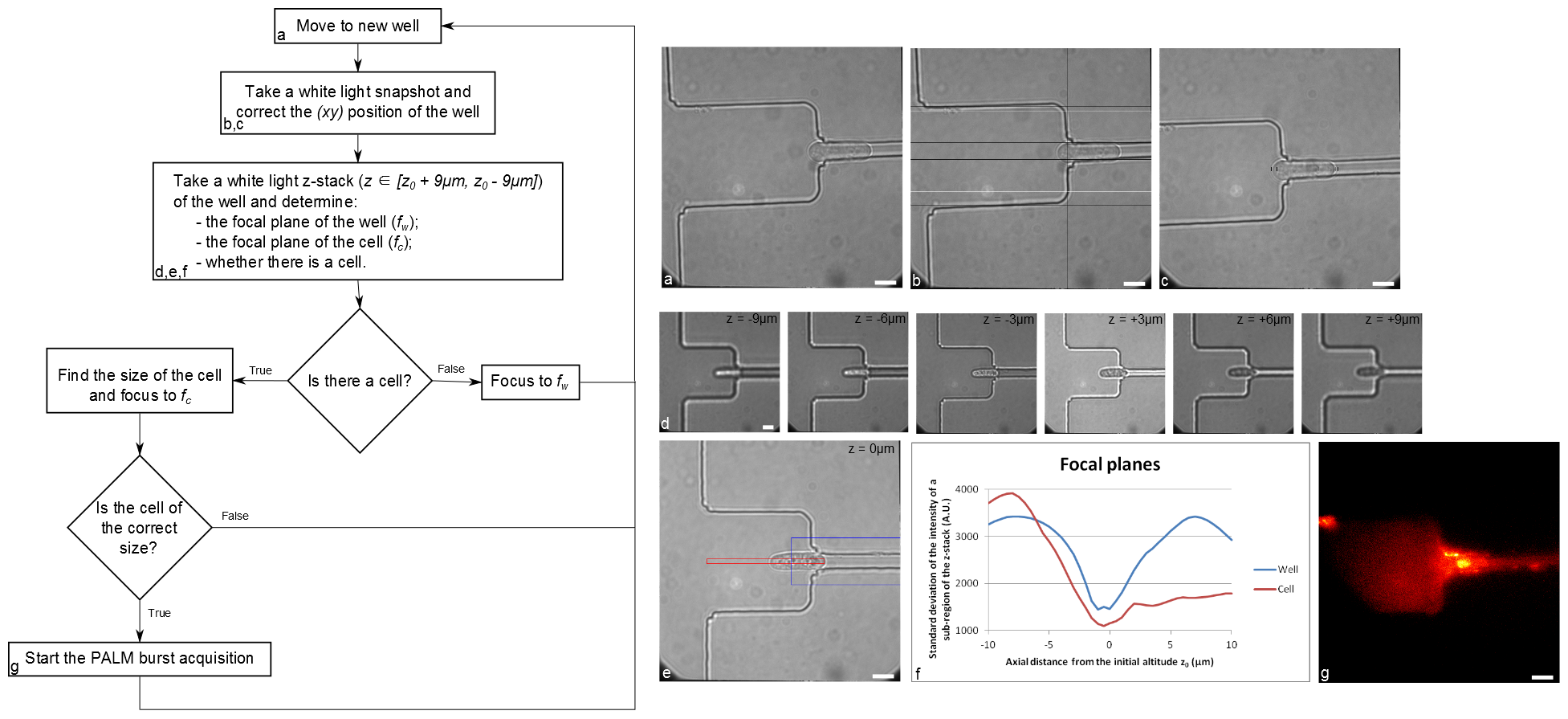


**Figure S-6:** Automation code. **Left panel**: flow chart of the automation algorithm designed for ‘smart’ large-scale data acquisition. **Right panel:** snapshots taken during the course of one representative cycle of the automation algorithm. For each cycle, the *(xy)* computer controlled microscope stage moves to the theoretical position of the new well, takes a transmitted light snapshot (a), analyses it by recognising characteristics of the borders of the well shown as fine lines (b) and adjusts the *(xy)* position of the stage to move the centre of the trap to the centre of the field of view and therefore the camera sensor (c). A transmitted light z-stack is then taken (d-e), allowing the determination of the focal plane of both the well and the possible cell by minimising the contrast (f) of both sub-regions of the well (blue rectangle, e) and of the possible cell (red rectangle, e). This analysis can be used to determine whether a cell has been trapped in the centre of the well and, if so, it finds its cell walls and length. Then, if a cell has been detected, the algorithm focuses the microscope onto its focal plane and starts a PALM acquisition (g: standard deviation projection of 50 frames of acquisition). Otherwise, it focuses onto the focal plane of the well before moving to the next well. **Scale bars**: 5 μm.

**Movie S-1:** Real-time demonstration of the automation algorithm. Some yeast cells were trapped in the device as previously described and imaged using the automation algorithm without its autofocusing step (Text S-1). First, the different steps of a cycle of the algorithm (Figure S-6) are presented and described. Then, a real-time movie screenshot of four cycles of the algorithm is played.

**Text S-1: Automation algorithm.**

**Principle:**

By knowing the position of the discreet sites where cells can be trapped, an automated microscope stage can visit and image many trapped cells. The aim of the algorithm is, once initially focussed on the first well in the first row of the device, to visit each trap site, determine if there is a trapped cell of a correct length of interest (i.e. stage of the cell cycle) and if so, perform a PALM experiment.

To account for the fact that both (1) the position of the trap site within the well varies along a row and (2) the axis of the trapping device may be at a slight angle relative to the detector, moving from one well to the next is done in two consecutive steps: firstly the *(xy)* stage moves to the theoretical place of the next well (as described in Figure S-1); then a ‘red light’ snapshot above 610 nm (to prevent any pre-activation of the fluorophores from light of wavelength below 610 nm, Figure S-6 a) is taken and binarised to detect the main characteristics of the well (four horizontal lines and one vertical one, Figure S-6 b); the position of the *(xy)* stage is finally adjusted to precisely place the centre of the trap in the centre of the camera chip (Figure S-6 c). In this way individual *S. pombe* cells always appear in the centre of the field of view independent of where the cell happens to be trapped.

For various reasons (stability of the optical components of the PALM platform, temperature or oil drift, non-orthogonal sample mounting, etc.), the focus can repeatedly drift on larger spatial scales (over more than five to ten wells). An autofocus function was thus added to the algorithm to correct for this instability: a ‘red light’ z-stack (Figure S-6 d-e) is taken to define the focal plane of the well and, if a cell is present, of the cell (which usually differs from the former, since the cell can be trapped at slightly different axial planes in the trap site). The autofocus analysis is based on contrast minimisation on sub-regions of the field of view over 9 μm above and below the initial *z* plane (see Figure S-6 e for the definition of the sub-regions: blue rectangle when looking at the focal plane of the well, red rectangle when looking at the focal plane of the possible cell; Figure S-6 f shows these regions for both the well and cell focal planes through minimisation of the contrast of the two sub-regions of interest). A trapped cell is detected if the contrast variation of the sub-region of the area of interest is greater than 1.5.

Then, if a trapped cell has been detected, its width and total length are measured. Finally, if the cell has a specific size (corresponding for example to the important but short-lived part of the yeast division cycle) it is imaged with PALM (Figure S-6 g). This ‘smart’ automation algorithm allows one to ‘search out’ and image a specific sub-population of cells from a large sample set while optimising the acquisition time, the required computer memory and the later analysis time.

**Design:**

The code is written in Java, using the ImageJ and Micro-Manager libraries. It is designed to run directly as a Micro-Manager plugin. To do so, the .class or .jar file should be placed in the ...\Program Files\Micro-Manager-1.4\mmplugins\ folder before loading Micro-Manager. The plugin starts when clicking onto "Microfluidic device STORM" in the plugin menu of Micro-Manager.

It runs with a specific hardware configuration, as follows:

- a red light shutter initially called "Shutter-1";
- an PALM imaging shutter initially called "Shutter-3";
- a camera which has a "MultiplierGain" property;
- a xy-stage;
- a z-stage with both "Set origin here" and "Set position Z (um)" properties.

It has been designed for a 512x512 camera chip with a pixel size in the image plane of 110 nm.

**Inputs:**

When started, the plugin opens a window asking for several parameters:

- The exposure time for the ‘red light’ snapshots (ms)
- The exposure time for the PALM burst (ms)
- The number of frames per PALM burst
- The minimum and maximum sizes for a detected yeast cell to be imaged with PALM (μm). (*-1* should be entered if no limit of size is wished)
- The time the stage should settle for after any *(xy)* movement (ms)
- The size of a pixel of the camera chip in the image plane (μm)
- The orientation of the trapping region of interest
- The name of the folder where the output files will be saved (spaces in the path provoke errors)
- The name of the ‘red light’ and PALM imaging shutters in the Micro-Manager local hardware configuration
- The name of the “set position” property of the *z* stage focus in the Micro-Manager local hardware configuration

**Outputs:**

A folder containing:

- The red light *.tif* file of each centred well
- The PALM *.tif* burst of each centred well containing a cell of the correct size
- A text file containing, for each well (defined by its row and well number):
  - - Its focal plane *f_w_*
    - The focal plane of its possible cell *f_c_*
    - The size of its possible detected trapped cell (*0* if none)
    - If a cell was detected, the number of cell walls measured and their *x* position in pixel
- A Temp directory containing details of the different steps of the image processing for each well (adjusting the position of the centre of the well, autofocusing)
